# Using radio programming to reach young adolescents with gender and sexual health information in a low-income urban setting in Kenya

**DOI:** 10.1186/s12978-025-01984-5

**Published:** 2025-05-31

**Authors:** Beatrice W. Maina, Vivian Nyakangi, Michelle Mbuthia, Caroline W. Kabiru

**Affiliations:** 1 African Population and Health Research Center, Nairobi, Kenya; 2 UNICEF Kenya Country Office, Nairobi, Kenya

**Keywords:** Radio intervention, Gender socialization, Adolescent sexual and reproductive health

## Abstract

**Background:**

Radio programs have been used to broadcast health information across Africa and beyond. However, there has been limited focus on radio programming targeting young adolescents (aged 10 – 14) with gender and sexual and reproductive health (SRH) information. An increasing body of evidence indicates a need to provide young individuals with accurate and easily accessible gender and SRH information to equip them to make well-informed choices about their SRH.

**Methods:**

We developed an engaging and educative seven-session radio show, which featured skits and guest speakers. A local radio station in Kenya broadcasted the show as a weekly episode over seven consecutive weeks. The main objective of the show was to improve young adolescents’ SRH knowledge, foster equitable gender norms, and enhance parent-adolescent relationships. To gauge perceptions about the radio program, we conducted in-depth interviews with a purposeful sample of 17 parents and 20 adolescents aged 12—14 years living in an informal settlement in Nairobi and who had participated in (or listened in on) at least three of the sessions; the radio manager and program presenter.

**Results:**

Both parents and adolescents indicated that they felt more connected to each other after listening to the program and this enhanced communication, especially on SRH issues. Resulting from the radio program, both adolescents and parents expressed greater awareness of gender and adolescent SRH issues, which were rarely discussed in detail in open forums in their context prior to the radio program. They recommended that such radio programs run regularly as they provide a platform where sensitive issues about adolescent health can be shared and discussed openly, allowing for both adolescent and community participation.

**Conclusions:**

Radio programming was perceived as a good platform for knowledge transfer and discussions about gender norms and SRH among young adolescents. However, messages should be designed to resonate with a diverse audience, as radio listenership will not only be limited to the target population.

## Plain language summary

Gender norms—acceptable and appropriate behaviors for men/boys and women/girls in a given community—are major drivers of health and wellbeing with strong implications for young people’s sexual and reproductive health (SRH) and behaviors. As young people transition through adolescence and adopt societal beliefs and systems, gender differences between boys and girls begin to manifest with far-reaching implications. Thus, interventions promoting equitable gender norms at an early age are essential in creating equitable societies and ensuring young people’s safe transition to adulthood. We undertook this study to explore adolescents' and their parents’ perspectives about a radio program (STaRS radio program) targeting young adolescents with gender and SRH information in an informal settlement in Nairobi. We interviewed 20 adolescents aged 12–14 years and 17 parents who had listened to at least three sessions of the radio program. The participants found the radio program acceptable and found it a useful platform for knowledge transfer and open discussions about gender and young people’s SRH. Additionally, the program elicited gender and SRH discussions between young people and their parents, which in turn enhanced parent–child relationships. For both adolescents and parents, the content of the program, which was holistic, inclusive, and collaborative, created an avenue to challenge inequitable gender norms. We conclude that radio programs can serve as low-cost interventions to reach young adolescents with gender and SRH information.

## Résumé

**Contexte:**

Les programmes radiophoniques ont été utilisés pour diffuser des informations sur la santé en Afrique et au-delà. Toutefois, les programmes radiophoniques ciblant les jeunes adolescents (âgés de 10 à 14 ans) et les informant sur le genre et la santé sexuelle et reproductive (SSR) ont suscité très peu d’intérêt. De plus en plus de données probantes indiquent la nécessité de fournir aux jeunes des informations précises et facilement accessibles sur le genre et la SSR afin de leur permettre de faire des choix éclairés en matière de SSR.

**Méthodes:**

Nous avons élaboré une émission de radio engageante et éducative en sept sessions, qui présentait des sketches et des conférenciers invités. Une station de radio locale au Kenya a diffusé l’émission sous forme d’épisode hebdomadaire pendant sept semaines consécutives. L’objectif principal de l’émission était d’améliorer les connaissances des jeunes adolescents en matière de SSR, de favoriser des normes de genre équitables et d’améliorer les relations parents-adolescents. Pour évaluer les perceptions au sujet de l’émission radio, nous avons mené des entretiens approfondis auprès d’un échantillon ciblé de 17 parents et 20 adolescents âgés de 12 à 14 ans vivant dans un quartier informel de Nairobi et qui avaient participé (ou écouté) à au moins trois des sessions.

**Résultats:**

Les parents comme les adolescents ont indiqué qu’ils se sentaient plus proches les uns des autres après avoir écouté l’émission et que cela avait amélioré la communication, en particulier sur les questions de SSR. À la suite de l’émission radio, les adolescents et les parents ont exprimé une plus grande sensibilisation aux questions de genre et de SSR des adolescents, qui étaient rarement abordées en détail dans des forums ouverts dans leur contexte avant l’émission radiophonique. Ils ont recommandé que de telles émissions radio soient diffusées régulièrement car elles offrent une plateforme où les questions sensibles sur la santé des adolescents peuvent être partagées et discutées ouvertement, ce qui permet à la fois la participation des adolescents et celle de la communauté.

**Conclusions:**

Les programmes radiophoniques ont été perçus comme une bonne plateforme pour le transfert de connaissances et les discussions sur les normes de genre et la SSR chez les jeunes adolescents. Cependant, les messages devraient être conçus de manière à trouver un écho auprès d’un public diversifié, car l’auditoire de la radio ne se limitera pas à la population cible.

## Background

Early adolescence, the age between 10 and 14 years, is a period of rapid physical, emotional, and social changes [1, 2]. Early adolescence is also a critical period for sexual maturation, acquisition of sexual and reproductive health (SRH) knowledge, and gender socialization [3, 4]. During this period, adolescents’ involvement in sexual behaviors can put them at significant risk for poor SRH outcomes [1]. At the same time, adolescence presents a window of opportunity for interventions that focus on positive adolescent development [3, 4].

The need to strengthen efforts to improve adolescent SRH has been increasingly recognized. Most recently, targeted inclusion of boys (and men) in addressing gender equality and adolescent SRH has been highlighted as of great importance in improving not only boys and men’s sexual health but also in addressing issues affecting girls (and women) [5, 6]. Adolescents in sub-Saharan Africa initiate sexual activity early [7], with evidence showing that non-penetrative sexual activities, such as kissing and touching, begin much earlier in early adolescence [8]. Some gender norms— acceptable and appropriate behaviors for men/boys and women/girls in a given community [9]—, especially those that portray sexual prowess among men and boys as a masculine trait and submissiveness and being subordinate as feminine [10] traits influence sexual behavior [11]. This evidence suggests the need to empower young people with accurate and accessible gender and SRH information enabling them to make informed SRH decisions.

Considering early adolescence is a key developmental stage in which behaviors are being molded, interventions should have a wide reach and should target a diverse audience including the proximal social networks such as parents that young adolescents engage with. Past studies show that interventions targeting young people with SRH information and services are diverse [12] and are often framed using ecological frameworks in reference to the different interactions, contexts, and influences young people have in regard to their SRH [10]. Findings from a study on gender norms and health showed that the complexity of gender norms and their intersection with other factors in the ecosystem can influence behavior across the life course [13]. The study showed that influences by parents and peers can have multiple and differing health consequences, gender norm non-conformity can influence negative sanctions, and the impact of gender norms on health can vary from one context to another [13]. Against these views, care should be taken when designing programs on gender and health, particularly on adolescent gender and SRH.

In the recent past, there has been an upsurge of SRH information shared through radio, internet, television, print media, and music, which has raised concerns about the exposure of adolescents to uncensored and age-inappropriate SRH information [14]. Despite these challenges, mass media interventions, including radio programs, are well-suited to deliver appropriate and inclusive interventions. Radio programs can serve as a low-cost form of communication with extensive coverage across geographical locations and populations and can be context-specific especially when community radio stations are used [15].

Radio programs have been used to broadcast SRH information for young people [15]. Several studies have found that mass media interventions can improve SRH outcomes and gender norms among adolescents [16–18]. However, there has been limited focus on radio programs specifically targeting young adolescents (aged 10–14) with gender and SRH information [19]. In addition, out-of-school adolescents are frequently excluded as many interventions tend to target school-going adolescents. A study conducted in rural southwest Uganda among out-of-school adolescents highlighted that different forms of SRH information should be delivered through different channels such as community educators, mass media, relatives, church, and community leaders [14]. Further, sexual health information and services for young adolescent boys are rarely included in most interventions [19]. This exclusion may limit boys’ ability to make informed decisions about issues that influence their lives across the life course.

Against this backdrop, we explored parents’, adolescents’, a radio manager’s and a presenter’s perceptions about a radio program tailored to address the needs of young adolescents in regard to gender norms and SRH in Viwandani, an informal settlement in Nairobi, Kenya. We sought to answer the question. “What is the relevance of radio programming in addressing gender norms and SRH issues among young adolescents in resource-poor urban settings?” The radio program was part of the study on “The gendered socialization of very young adolescents and sexual and reproductive health (The STaRS project)” [20, 21]

## Methods

This study aimed to explore perceptions towards using a radio program to broadcast gender and SRH information relevant to young adolescents.

### Study design

To gauge the audience’s and program presenter’s perceptions and views about the radio program, we conducted a post-intervention qualitative study comprising in-depth interviews with adolescents, parents, the radio station manager, and the program presenter.

### Study site

Viwandani is a resource-poor setting in Nairobi that is covered by the Nairobi Urban Health and Demographic Surveillance System (NUHDSS) and is located about 12 km from Nairobi’s city center [22]. As of 2018, Viwandani had a total population of 52,698 covered by the NUHDSS with slightly more than seven percent being adolescents aged 10–14 years [23]. Viwandani is generally characterized by abject poverty, high levels of unemployment, substandard and overcrowded housing, limited education and social services, high levels of crime and insecurity, inadequate water and sanitation infrastructure, and exposure to risky sexual behaviors among young people [22, 23]. The major source of livelihood in these settlements is casual employment. Viwandani has a relatively youthful and highly mobile population seeking job opportunities in the nearby industries.

### Participants

We held interviews with a purposeful sample of 20 young adolescents (9 females and 11 males) and 17 parents (15 females and 2 males). Adolescents and parents were selected if they had participated in (or listened in on) at least three of the seven radio sessions. We also considered age and sex balance when selecting adolescents. We used the phone database of two schools that participated in the formative study to contact and identify eligible adolescents and parents. The two schools were identified through a school mapping exercise to identify which public-day primary schools a majority of learners in the Viwandani informal settlement attended. In addition, we interviewed the station manager and the program presenter, who were both males, to explore their perceptions and experience about broadcasting sensitive topics. According to Hennink (2022), our sample is deemed fit to reach data saturation in qualitative studies [24].

### Data collection

To guide the interviews, we developed a semi-structured interview guide that included questions on adolescence, adolescent SRH, gender socialization, parent–child connectedness, and participants’ views of the radio program. Interviews were conducted by trained research assistants at the field office of the lead implementing partner. The offices offered a central and private space to conduct interviews. Only the interviewer and the interviewee were allowed in the interview rooms during the interview. Interviews were held in either English, Swahili, or a mix of both languages based on the participant’s preference. Interviews were also audio-recorded with consent and assent, as applicable, from the participants.

### Data analysis

The audio-recordings for interviews conducted in English were transcribed verbatim. For interviews conducted in Swahili or a mix of Swahili and English, audio-recordings were directly transcribed into English by bilingual transcribers. To ensure the accuracy of the transcription and translation, the authors randomly selected and cross-checked half of the transcripts with the audio-recordings. The transcripts were then uploaded into NVIVO and analyzed using inductive thematic analysis. For this study, we focused on themes around participants’ views of the radio program.

All transcripts were read by the lead author, identifying a set of codes emerging for each theme and checking for emerging patterns, commonalities, and differences under each theme. The codes were then discussed by all authors, resolving any discrepancies identified.

### Fieldwork training

Four qualitative research assistants (two male and two female) were recruited and attended a three-day fieldwork training. The training covered topics on: study overview, objectives, and methodology; overview of the radio program; human research ethics; interviewing techniques; key considerations while conducting interviews with vulnerable populations, including very young adolescents and populations living in informal settlements; informed consent and consenting process; and review of the interview guides. Mock interview sessions were held to gauge the interviewers understanding of the study concepts.

### Ethical considerations

Ethical approval was obtained from AMREF Health Africa Ethics and Scientific Review Committee (Ref.AMREF-ESRC P564/2018) and a research permit from the National Commission for Science, Technology and Innovation (Ref. NACOSTI/P/19/16027/27880). Research assistants were trained on ethical practices while conducting research with human participants and signed confidentiality agreements. Parental consent and adolescent assent were obtained prior to conducting the interviews. Given that the research occurred during the COVID-19 pandemic, research assistants adhered to institutional and national COVID-19 prevention protocols.

Below we briefly describe the radio program. Firstly, we detail the context of the intervention and the design of the radio program, and then we share findings from qualitative interviews showing the relevance of the radio program to the study community.

### The context

The radio program was developed using a broad evidence-based approach. Firstly, a formative survey was conducted to examine young adolescents’ sociodemographic, family, school, and peer characteristics, access to SRH information, sexual behavior, gender attitudes and beliefs, among other aspects [20]. Secondly, a community dialogue approach was used to validate the formative study findings and to discuss opportunities for interventions specific to the study context [25]. The aim of the community dialogues was to identify gaps in gender and SRH programming among young adolescents and to co-develop interventions to foster equitable gender norms and improve adolescent SRH outcomes. The meetings brought together about 40 participants, including adolescents, parents/caregivers, teachers, the community advisory committee (a group of community leaders representing different sub-populations and who serve as the community gatekeepers), researchers, program implementers, and other stakeholders working on gender and adolescent SRH issues within the study setting.

### The radio program

**Fig. 1:**
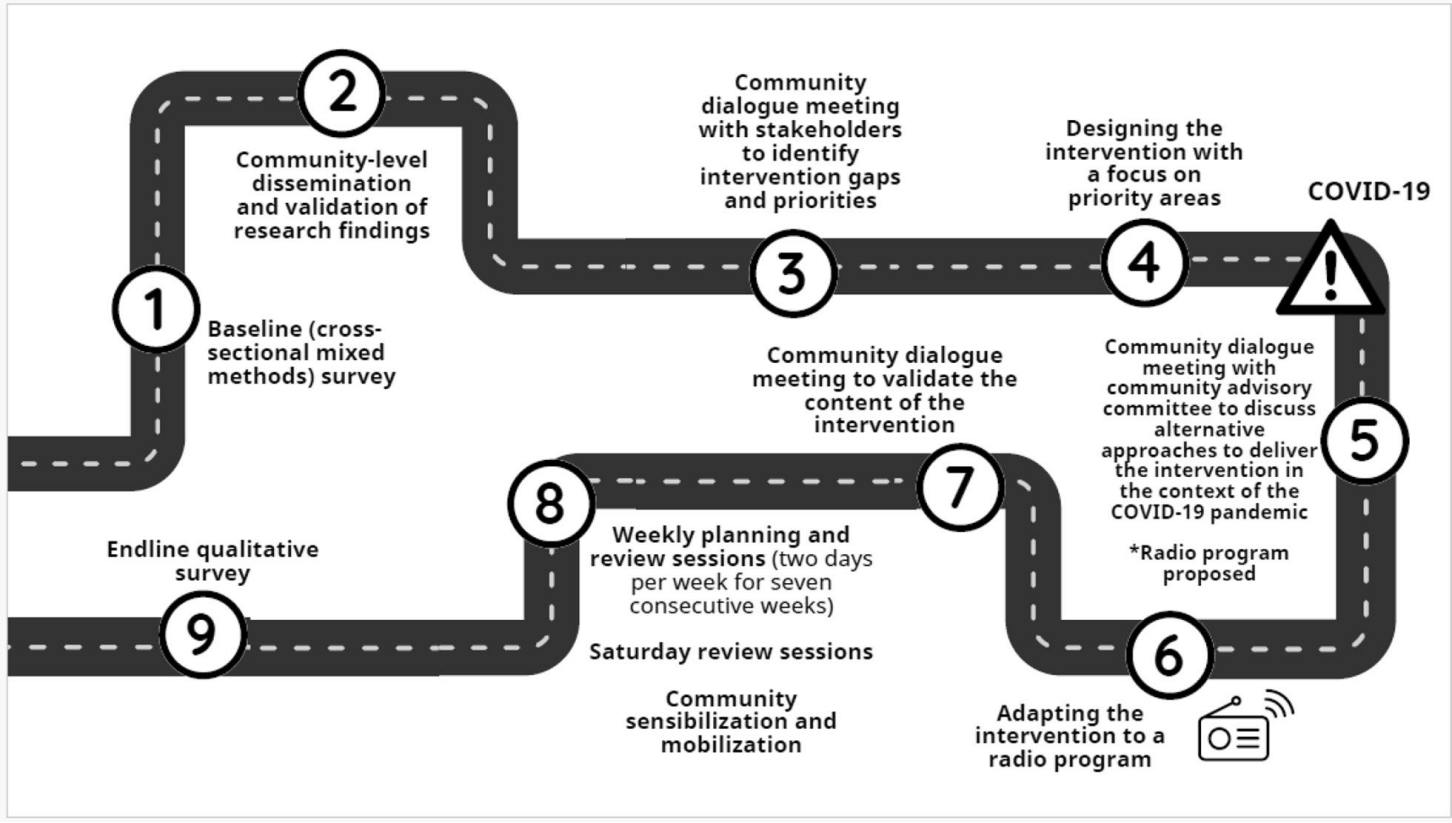
Intervention development and feasibility assessment process

While the initial plans were to have school-based, face-to-face interventions, it was impossible to do so in the context of the COVID-19 pandemic. Through a community dialogue meeting with the community advisory committee, we identified radio programming as an alternative and the most appropriate approach to reach young adolescents considering the precautionary measures put in place to stop the spread of the Corona virus, including the closure of schools. Figure 1 describes the sequence of activities involved in the development, implementation, and assessment of the radio program.

The research team worked with a community-based organization, U-Tena (https://www.u-tena.org/), and a community-based radio station, Ruben FM (https://rubenfm.or.ke/), to design and implement a radio program called the “STaRS Radio Program” [21]. The radio program consisted of seven sessions, which were broadcasted as live shows within seven consecutive weeks, one-hour per week (on Saturday), for a period of two months (December 2020 to January 2021). The sessions followed the same format and were primarily facilitated by peer mentors from U-Tena and moderated by the radio presenter from Ruben FM. In four of the sessions, guest speakers, with professional expertise on specific topical issues and who were working within the community, were invited to offer insights as shown on Table 1. The experts were identified through U-Tena.

**Table 1:** Summary of the radio sessions*

Session	Topic	Focus	Facilitators
1	Ensuring a gender equitable society	• *Gender inequalities;* • *Enhancing equitable gender socialization process for young adolescents*	Two peer mentors (Male and female)
2	Adolescence period and gender socialization	• *Adolescence transition: Pubertal changes and associated risks and opportunities;* • *Gender expectations during adolescence*	One Peer mentor (Female) One Medical Practitioner (Male)
3	Adolescent sexual and reproductive health	• *Sexual and reproductive health knowledge and information sources* • *Risky sexual behaviors, associated pathways and outcomes* • *Sexual violence/abuse: Knowledge; risk and protective factors; reporting avenues*	One Medical practitioner (Male) One Peer mentor (Female)
4	Parent–child connectedness	• *Cultivating an environment that enhances parent–child communication in general, and specifically on SRH issues*	Two Peer mentors (Male and female)
5	Effective child monitoring and supervision	• *Child safety and protection*	One peer mentor (Male) One social worker (Female)
6	Peer relationships	• *Identifying, enhancing and maintaining good peer relationships*	Two peer mentors (Male and female)
7	Future aspirations and empowerment	• *Identifying career progressions, aspirations and empowerment for young adolescents*	One teacher (Male) One peer mentor (Female)

^*^Detailed description of the sessions can be found on the study report [21]

The broadcasting period was strategic to when adolescents were most likely to be available without interference with learning activities. Although at the start of the radio program, schools were closed due to the COVID-19 pandemic, schools are generally closed for holidays in December. Saturday was also strategic, as schools are not in session on weekends and more parents would be home from work. The sessions were broadcast in a mixture of English and Swahili, languages that are commonly used and understood by young adolescents and parents in that setting. The intervention was aired through Ruben FM, which broadcasts within a three-kilometer radius of the study community. In consideration of young adolescents’ developmental stage [26], a participatory edutainment approach was used to deliver key messages around the seven topical areas as shown on Table 1. Though the main target group was young adolescents, the intervention was holistic in that it engaged parents and the wider community as well.

Owusu-Ansah and Oforiwaa Mensah (2014) argue that communication is a two-way traffic involving the sharing of ideas, knowledge, and experience [14]. In designing the intervention, we ensured that young adolescents, their parents, and the community could call in and ask questions via a toll-free number, as well as engage via social media platforms and text messaging. While we worked with trained peer mentors from U-Tena to deliver some life skills sessions, we also brought in experts on gender issues and adolescents. The experts were people working within the community and had contextual knowledge of how young adolescents live. To elicit discussions around topical issues, we developed skits informed by the day-to-day life experiences of young adolescents in the informal settlement. The skits followed the lives of hypothetical girls and boys in the study setting and the gender and SRH issues they encountered or experienced. Through the skits and the open dialogue channels, we ensured communication between the adolescents and the agents of the messages.

As part of community engagement, and to maximize participation among young adolescents, village leaders visited each household with young adolescents to remind them of the forthcoming session. In addition, text messages were sent to the parents a day before each show as a reminder to listen and allow their young adolescents to listen to the radio program.

## Results

### Characteristics of study participants

Table 2 presents the characteristics of the study participants.

**Table 2:** Sociodemographic characteristics of young adolescents and parents*

Characteristic	Adolescents (absolute number)	Parents (absolute number)
Age		
12	8	
13	7	
14	4	
Not specified	1	
Mean age (SD)		47.5 (5.2)
Sex		
Female	9	15
Male	11	2
School grade		
Primary 6	8	
Primary 7	11	
Not reported	1	
Living arrangements		
Both parents	8	
Mother only	9	
Father only	1	
Other relatives/not reported	2	
Ethnicity **		
Kikuyu		6
Kamba		4
Luhya		3
Luo		2
Other		2
Occupation		
Employed		8
Self employed		4
Not employed		5
Number of children		
1 – 2		6
3 – 4		8
5 or more		3
Total	20	17

^*^We also interviewed one radio station manager and the radio presenter hosting the STaRS radio program; both were male; age data were not captured

^**^We did not collect ethnicity data from adolescents

Below we present findings about participants’ perceptions on the acceptability and relevance of the STaRS radio program. We describe participants’ perceptions about the radio program and categorize them into three thematic areas: program design and messaging, content appropriateness/relevance, program outcomes.

Generally, we did not observe key differences in responses provided by male versus female adolescents, and so findings are presented without any attribution to gender. Similarly, there were only two male parents interviewed compared with fifteen female parents. This imbalance does not allow for a comparative analysis of findings. However, where possible, we have presented quotations from both male and female parents.

### Program design and messaging

The radio program was said to be acceptable and appropriate in the study setting as it addressed pertinent community issues related to young adolescents. The program was also termed holistic as it not only targeted issues affecting young adolescents, but was also designed to engage with the whole community.

This radio program is good because it teaches people, children, and adults. They get information (adolescent girl, AG01)

About the content, I also felt it, because the content was mainly targeting young adolescents but during the airing most of the parents felt the content engaged them; it cut across all young people, and through listenership and feedback, you can gauge it feels the program really had an impact on the listeners (radio presenter)

The community is very positive about it [the program], and when you do something and you see people keep on asking “will you continue having it” [the program], this means it was of great impact to them and they have received it positively. As a community radio station, we really need to create that good rapport with the community, and our work is simply to educate, entertain, and inform and if you ensure that you have right content like educating them on the right issues that are actually affecting them, they will stick to the radio and listen to it so I can say very positive response from the calls, SMS, and social media interactions (radio manager)

In addition, the program was inclusive as it addressed issues concerning both boys and girls, which was termed a rare occurrence in adolescent programming where boys are often left out.

Most of the programs focus on girls, they should also focus on boys. These programs should treat all of us equally and have programs like this one for boys and girls [STaRS radio program] or like the way we are taught in school. All of us are equal, so when we are taught, we will all get knowledge because there are some girls who are ignorant and they would have sex with boys and they get pregnant, but it would not be the fault of the boy since the girl was taught while the boy was not (adolescent girl, AG05)

Participants highlighted that they were allowed to participate and air their views through call-in sessions, allowing for greater community engagement through open discussions.

They had good teachings, even the parents contributed to the topics that were being discussed on the radio through making phone calls (female parent, FP02)

This program will improve many people because there are others in school who listen and come to tell me that it has made them feel nice; even others said Ruben FM is good. It was my mother who was switching on the program for me. I even knew the frequency of Ruben FM is 99.9 and they were loud and clear. I had a question during one session, and my mother gave me her phone to call the radio station and ask. So many people were also calling to ask or answer questions (male adolescent, AB06)

The community-based radio station (Ruben FM) aims to empower listeners with information affecting their health and wellbeing, in addition to providing up-to-date news and entertainment. The STaRS radio program aligned with this aim by providing information and eliciting discussions on neglected but topical issues addressing the needs of young adolescents. The radio manager noted that designing the program and collaborating with those having expertise in specific areas enhanced the radio program.

I think it was an added advantage to us because I am just a journalist and my profession is journalism, but now interacting with somebody who has expertise and talking to the kids and also helping them to get solutions to some of the challenges that are affecting them is an advantage to us, so it was one of the best projects we have had at Ruben FM (radio manager)

The radio manager also noted that the teamwork demonstrated during content generation was a valuable lesson for others, explaining that preparation, through intense research and consultation with experts, enhanced the quality and fluency of discussions during radio broadcasts.

While acknowledging the relevance of the content delivered through the radio program, participants expressed mixed reactions on whether similar interventions could be delivered within school settings. On one hand, some participants felt that if such a program were held in school settings, it could reach a wider group of adolescents, including those with no access to radio. On the other hand, others felt that it would be difficult to have discussions on sensitive issues like SRH in school settings and that the discussions would exclude the wider community that is outside the school setting, therefore lacking inter-generational dialogues. Stigmatization in schools was also a concern, with some participants indicating that having the intervention in a school setting would limit active participation.

School-based programs may limit participation for fear of saying something wrong and being laughed at or bullied thereafter (adolescent girl, AG19)

### Content appropriateness and relevance

This theme focused on participants views about the content aired on the radio program. Overall, the content was said to be acceptable and appropriate not only for the targeted audience but also for the community as a whole.

Both adolescents and parents perceived the radio program as a means of gaining information about gender norms and SRH issues affecting young adolescents. The majority acknowledged having discussions on adolescent SRH on radio was a game changer for them, as such discussions were often considered taboo. In addition to learning about the physical, biological, social, and cognitive changes that happen in early adolescence through the radio program, participants were able to reflect on the risk factors associated with poor adolescent health and behavioral outcome within their environment and to identify ways to overcome these risk factors.

I was told by my mother when I was young that adolescence is something bad, but now, after listening to the program, I know adolescence is a stage. There are things one should do and there are those you should not do, like having sex (adolescent boy, AB11)

When a person reaches adolescence, some start to get angry very fast, and they are disturbed by adolescence, which also controls them, and girls start hanging with boys and arguing with their parents. There are some girls while at home when they are beaten, even if it is softly, they go to sleep with their boyfriends. Their mothers will come to realize this when their child gets pregnant or infected with a disease. Now, this program has taught us how to deal with such problems and even how to overcome challenges that come with adolescence (adolescent girl, AG14)

For some parents, listening to the radio show helped them reflect on their experiences growing up, the challenges they faced as they transitioned through adolescence, and how these experiences and challenges had shaped their current life. Parents also reported that listening to the radio program gave them information on how to guide their children in navigating the risky environments within the informal settlements. Other parents described how the shows helped them understand why their adolescents behaved in a certain way (for example, being aggressive and having mood swings), which enabled them to be less judgmental.

My perception about adolescents has changed because before I used to be harsh to my child, I was even insulting her, and also, I used to compare her with her friends. For example, I may ask her why she is asleep while her friend is fetching water and they were together before. So, when I listened to the program, I learnt that I am not supposed to compare my child with another child since I would be hurting her and she may be going through some stuff. So, it is better I tell her directly what I want rather than comparing her with someone else. So, I learnt I should not harass her because this stage is very hard to control. (female parent, FP16)

The important thing I learnt is that, when a child reaches this stage, sit down with them and start telling them what is good and what is bad. The way you train your child when they are young is the way they will be when they grow, and when you raise your child by instruction, their character will be good (male parent, MP012)

Participants also felt that the content was timely as it addressed COVID-19 related concerns.

Last year, COVID-19 was a big challenge, especially for girls staying at home together with boys, parents and community at large. There were so many issues that were coming into the radio station through the calling sessions that we used to have. People complaining that children are being molested; parenting was also an issue, and teenage pregnancies were a real big issue, but when this program came in, it helped us to address these issues because there were issues happening in the community at that time (radio manager)

COVID-19 has affected both boys and girls, but girls more because some have gotten pregnant and dropped out of school. They have grown big, so they fear going back to school as they will be laughed at. If they listened to this program, it has taught them what is right and wrong, they won’t repeat (adolescent boy, AB09)

When COVID-19 cases were high it was hard task for parents, for example, I used to stay with the girl who was following this boy and she also listened to the program, and when she went out, I asked her where she was going, and it was a must for me to know where she went. It was a hard task protecting the children all the days, and I think it was almost a year, but the radio program and the television helped since there were some programs which they watched (male parent, MP05)

### Program outcomes

Based on the content of the radio program and the discussions that were embedded in each session, participants acknowledged the existence of unequal gender norms in their setting. This not only reflected on the sharing of household responsibilities but also on how gender norms impacted on young adolescents’ SRH behavior and shaping their life trajectories.

…You should tell them that a person is supposed to have sex when they reach a certain age and also that it is bad to have sex. Having sex can ruin their life, and they can get infected with diseases like HIV and STIs. Also, girls can get pregnant, ruin their lives and fail to achieve their dreams (female parent, FP01)

Participants highlighted how the program played a role in challenging inequitable gender norms. Young people were said to copy what they see within their environment, most of which were gender inequitable norms. The discussion on inequitable norms on the radio program enabled parents and adolescents to reflect on their own gender attitudes and ways in which gender norms influenced their own experiences.

About the tradition that girls are the ones to wash dishes; boys are not to wash. You can have a sister who is still too young to do the washing, so you will have to wash. There is no work for a boy and for a girl because they are the same. (adolescent boy, AB09)

Another aspect of the program outcome was the improved communication between adolescents and their parents. Some parents and adolescents noted that prior to listening to the radio show they had never had conversations about SRH with their adolescent children and parents, respectively. For some, the radio program acted as a conversation starter, enabling deeper conversations about SRH and gender norms. Some parents explained that listening to the radio show with their children helped create the rapport they needed to initiate such conversations.

In the STaRs program, we were taught a lot that we had not been taught in school. Now we discuss with my mother. It just started with questions: how I am, whether I have ever gone through those things outside there [the content of the STaRs program], like being abused sexually by someone I do not know, whether I have ever met a stranger and got kidnapped, whether I have had a girlfriend. Because we get along well with my mother, after the program we talk, I answer her, and she also answers me. (adolescent boy, AB06)

My child has started to be open to me, even, she told me that she can’t get pregnant, she would maintain herself well, and she would not play with boys. I have seen that she has changed, and she started to have self-awareness. I would be available to my daughter although our parents did not have time for their children because they were busy on their work, but in my part, it is a must to stay with my child so that she would not blame me later that there is something I did not tell her (female parent, FP17)

In addition, using skits to mimic real-life experiences of adolescents in the study context enhanced discussions as illustrated by the radio host:

About the skit, you may find that it mainly introduces what the presenter and also the guest, like doctors were going to discuss. When the listeners heard the skit most of them felt a connection and started participating before we even asked the questions. I can say that the skit brought them together and caught their attention. The guest speakers brought in content aligned with the topic of the day, and so listeners were able to engage by asking questions or discussing. I felt that the listeners were feeling more engaged. (radio presenter)

Similarly, parents and adolescents reported improved parent–child relationships. Some parents indicated previously feeling disempowered to discuss some gender and SRH topics with their adolescent children and avoided having conversations. Some adolescents going through pubertal changes reported that prior to the radio show they felt misunderstood and disengaged from their parents.

Before the radio program, I did not relate well with my mother. She could just give me stories and warnings. Now she teaches me. We understand each other. (adolescent boy, AB11)

The last time I listened to it, they said that we should interact with our parents, we should talk to them more, if we feel difficult in anything we should also talk to them and they would see how to solve it. Because my mother listened to it and she told me to tell her if anything wrong happens to me. She also told me if anyone abuses me sexually I should tell her because she knows how to deal with it, so I knew my mother will understand anything if I tell her (adolescent girl, AG05)

Overall, our findings show that radio programming is an acceptable channel to engage with communities on gender and SRH issues, more so, issues affecting young people. This allows for a wider reach and range of topics being discussed in open forums as well as allowing for community participation. Despite this, the study was not without challenges that need to be taken into consideration when designing such interventions. First, structural barriers such as loss of electricity and internet connectivity affected listenership. This can be corrected by having a repeat of the recorded sessions, unlike in our study, where each session was aired only once. Second, the presenter’s ability to connect with listeners and create a space for open dialogues highly contributes to the success of the show. In our program, the radio presenter had experience hosting edutainment shows for young people, which made it easy for him to create rapport.

## Discussion

This study explored perceptions towards a radio program aimed at addressing gender norms and SRH among young adolescents. The findings show that the radio program was acceptable and the content was appropriate within the study context.

Lack of access to SRH information and existing social norms in different contexts have contributed to SRH risks for young people, such as increased number of lifetime sexual partners, inconsistent or non-use of condoms, multiple sexual partners, sexual violence, unintended pregnancies, and sexually transmitted infections [27]. Similarly, studies have shown how unequal gender norms are risk factors for poor SRH outcomes. Access to information about SRH and equitable gender norms during early adolescence, a critical developmental stage, can help young adolescents make informed SRH choices. Mass media can be an important source of SRH information for young people.

Both adolescents and their parents underscored the importance of the STaRS radio program in sharing information and enhancing discussions on sensitive topics that are barely talked about not only at the community level but also amongst parents and adolescents [28]. Such issues include sexual development and associated risk factors in early adolescence, parent and peer relationships, child safety, and empowerment [29], as well as societal norms and gendered socialization [3]. Adolescents indicated learning the importance of identifying and avoiding the risks they face and discussing SRH issues with their parents. Parents reported increased awareness of adolescent development and associated risks, as well as the need to communicate openly and freely with their children, especially on gender and SRH issues. Both parents and adolescents indicated they felt more connected to each other after listening to the radio program, which in turn enhanced communication.

The debate on the exclusion of boys in gender and SRH programming has been ongoing [30, 31]. Ruane-McAteer and colleagues (2020) argue that it is imperative to include boys and men in programming, and the basic question should be “how to do so?” while promoting gender equality and health for all [30]. The STaRS radio program was cognizant of the need to include boys and men in all topics covered by the program. Both male and female adolescents appreciated the content of the program, and our results did not show any gender differences in adolescents’ perceptions towards the radio program. While we note the scarcity of literature on interventions targeting early adolescents, moreso in our study context, Widman and colleagues [32] found similar findings when evaluating the feasibility, acceptability, and preliminary efficacy of a 45-min interactive, online sexual health program for adolescents, called Health Education and Relationship Training. In the STaRS program, adolescents and parents acknowledged that the program helped them recognize that both boys and girls should be treated equally, emphasizing gender equality in all aspects. Consistent with existing literature [33], parents noted that there had been limited attention to SRH issues affecting adolescent boys and men at the household and community levels and commended the radio program for highlighting boys’ vulnerabilities and the corresponding risks they are frequently exposed to. This finding underscores the need for greater engagement of boys as agents of change in enhancing equitable and healthy societies. The need to involve boys may be more urgent in patriarchal societies where men are often seen as the perpetrators of unequal gender norms. While our study findings demonstrate the value of radio programming in reaching adolescent boys at community level with context-relevant information on gender and SRH, we are cognizant of the fact that a majority of male parents did not participate in the intervention. Other studies have reported similar challenges with engaging men in gender and SRH intervention [34]. Despite this, early adolescence presents an opportune period for gender and SRH interventions [12].

Community dialogue and engagement were key components of the radio program, from design to implementation and evaluation. According to Kesterton and Cabral de Mello (2010), community engagement is useful in generating demand and support for young people’s SRH issues, including access to information that is culturally sensitive [35]. Studies that do not engage with the community right from the start may fail to capture key and culturally acceptable issues that the communities need addressed. In this study, the parents viewed community dialogues as an approach to highlight key issues affecting young adolescents and suggested approaches on how these issues can be addressed for individual and collective change. Further, the interactive engagement during the community dialogues and the radio program’s live sessions led to active participation and open discussions about gender and SRH issues. As argued by Trickett et al. (2011), community engagement places the community capacity as a central concept, shifting the focus from needs and deficits to strengths and resources to build on [36]. Such resources include community participation in program design and use of community-based organizations and networks, including local radio stations, available skills, and knowledge leadership [37].

Another key aspect of radio programming is how key messages are packaged and delivered. In designing and implementing effective programs, it is important to assess the communication messages used and ensure they are culturally acceptable [14]. By using hypothetical skits mimicking real-life situations for adolescents in the study settings, we were able to elicit discussions and encourage open discussions as listeners related to the scenarios aired. The design and the messaging allowed the listeners to critically analyze the situation at hand and look for possible solutions, thus, making the listener a core decision-maker, exerting agency on issues affecting their life and being a primary agent of socialization [14, 38].

While we note the acceptability of the radio program in the study setting, we are cognizant of the complexity of gender norms and the interactions within an ecosystem that influence multiple and differing behaviors with implications across the life course. Thus, there is a need to target other aspects at all levels of the ecosystem to ensure consistency. Similarly, we recognize that non-conformity to some gender norms can lead to social sanctions with implications for health and wellbeing [13]. Thus, extreme care should be taken when designing interventions to ensure they do not cause harm to the participants.

The merits of the radio program as discussed by the participants show it is a useful tool to share information on gender, health, and wellbeing of young adolescents, targeting the adolescents, their parents, and the community at large. The world has experienced an upsurge of community radio stations in the recent past, as shown by World Radio Map [39]. This has contributed to not only changes in the social landscape but also the dialogic nature of conversations held within specific communities across geographical, cultural, social, and faith-based affiliations, among others. Leveraging on these contexts, radio programming can be a useful tool to pass information that is contextually relevant, packaged for specific sub-populations, and in languages understood by a majority. Radio programs enhance inclusion, allowing for open generational and intergenerational dialogues as well as empowering communities by giving them a platform to challenge gender inequalities, norms, and attributes they deem unfit.

## Limitations

Study findings should be interpreted in light of several limitations. First, because of the change in the intervention design due to the COVID-19 pandemic, we were unable to evaluate whether there was a “real” change in participants’ knowledge, attitudes, and practices as a result of listening to the radio program. Second, we had no way of monitoring participants’ engagement with the program and thus relied on self-reported data that could be subject to social desirability bias. Additionally, local radio stations may not have the capacity to track listenership other than those listening livestream, causing under- or over-reporting. Nonetheless, triangulating information from adolescents, parents, and the radio team’s perceptions adds to our confidence in the results. Third, it was challenging to find male parents, as they did not meet the minimum threshold to participate in the study. Thus, the study findings from parents mainly reflect perceptions from female parents.

## Conclusion

Mass media interventions, including radio programs, can serve as a low-cost intervention to reach a diverse audience with critical health information. In this study, we found that radio programming was considered a good platform to widely share information on gender norms and SRH among young adolescents. We found that parents and adolescents reported that listening to the radio program enabled them to have more open discussions about gender and SRH issues, strengthened parent–child communication, and enabled them to reflect on their gender attitudes and practices. However, there are critical considerations while implementing radio-based interventions. First, radio programs have a diverse audience. As such, key messages should resonate with a diverse audience even where the major topics relate to a specific population, such as young adolescents. Second, the content delivered via a radio program is highly summarized, and there is a need to ensure continuity of discussions beyond the radio program. Having community ownership of the program is likely to ensure sustainability; however, such continuity and sustainability in poor-resource settings is limited by a lack of resources, such as funding to run such program.

## Data Availability

Data and materials are available upon request from the APHRC’s micro-data portal (https://aphrc.org/microdata-portal/).

## Abbreviations

SD: Standard deviation
SRH: Sexual and reproductive health
STaRS: Starting Right at School
